# Evaluation of continuous quality improvement in accreditation for medical education

**DOI:** 10.1186/s12909-020-02124-2

**Published:** 2020-09-28

**Authors:** Nesibe Akdemir, Linda N. Peterson, Craig M. Campbell, Fedde Scheele

**Affiliations:** 1 OLVG Teaching Hospital, Amsterdam, the Netherlands; 2 Amsterdam UMC, Amsterdam, the Netherlands; 3 Committee on Accreditation of Canadian Medical Schools, Ottawa, Canada; 4 Royal College of Physicians and Surgeons of Canada, Ottawa, Canada; 5 Athena Institute for Transdisciplinary Research, Amsterdam, the Netherlands; 6 Dutch Royal Medical Council, Chair Legislative College for Accreditation of Residency Training 2016–2019, Utrecht, the Netherlands

**Keywords:** Accountability, Accreditation, Continuous quality improvement, Internal review, Medical education, Quality management

## Abstract

**Background:**

Accreditation systems are based on a number of principles and purposes that vary across jurisdictions. Decision making about accreditation governance suffers from a paucity of evidence. This paper evaluates the pros and cons of continuous quality improvement (CQI) within educational institutions that have traditionally been accredited based on episodic evaluation by external reviewers.

**Methods:**

A naturalistic utility-focused evaluation was performed. Seven criteria, each relevant to government oversight, were used to evaluate the pros and cons of the use of CQI in three medical school accreditation systems across the continuum of medical education. The authors, all involved in the governance of accreditation, iteratively discussed CQI in their medical education contexts in light of the seven criteria until consensus was reached about general patterns.

**Results:**

Because institutional CQI makes use of early warning systems, it may enhance the reflective function of accreditation. In the three medical accreditation systems examined, external accreditors lacked the ability to respond quickly to local events or societal developments. There is a potential role for CQI in safeguarding the public interest. Moreover, the central governance structure of accreditation may benefit from decentralized CQI. However, CQI has weaknesses with respect to impartiality, independence, and public accountability, as well as with the ability to balance expectations with capacity.

**Conclusion:**

CQI, as evaluated with the seven criteria of oversight, has pros and cons. Its use still depends on the balance between the expected positive effects—especially increased reflection and faster response to important issues—versus the potential impediments. A toxic culture that affects impartiality and independence, as well as the need to invest in bureaucratic systems may make in impractical for some institutions to undertake CQI.

## Background

The design [1], as well as the value of accreditation systems [2, 3], are topics that require additional research. In this study we reflect on the virtues of continuous quality improvement (CQI) in medical education.

There is an implicit belief that the accreditation of medical education programs ensures the quality of the education these programs provide and the graduates they produce [4–6]. The importance of the accreditation of medical education programs has been further heightened by a recent announcement by the Educational Commission for Foreign Medical Graduates. The announcement states that, by the year 2023, the only candidates who can be certified, and therefore considered for residency education in the United States, will be graduates of medical schools that have been accredited by organizations approved by the World Federation of Medical Education as working to appropriately formulated and applied standards [7]. Despite the respect that accreditation is accorded, little has been written about whether the accreditation process actually achieves its intended outcomes. In many jurisdictions, accreditation of medical education programs has traditionally been based on episodic evaluation [8]. In general terms, an episodic evaluation is an external review by an accreditation authority that includes on-site visits and is repeated at specified intervals [8, 9]. More recently, the focus of accreditation has been shifting from episodic evaluation to CQI [10–13]. CQI, which originated in industry as an approach to quality control for services or products [14–17], is a never-ending cycle of organizational improvement that uses quality indicators [18].

This paper, one of a series arising from the first World Summit on Accreditation Outcomes in Medicine, explores the role of CQI in systems traditionally based on episodic evaluation. As explained before, the evidence in literature for the benefits of a CQI system in medical education is insufficient, although CQI has been introduced in several jurisdictions. The result is a new bureaucratic instrument that is being used without sufficient empirical evidence. As such, it is important to investigate the pros and cons of CQI in medical education. In this utility-focused evaluation [19] we use a set of quality criteria for governance oversight to shed more light on these pros and cons.

## Methods

We chose to perform a naturalistic utility-focused evaluation [19]. Three of the authors (LP, CC, and FS) were responsible accreditation officers in their distinct fields of medical education who wished to add to the evidence and inform decision making around investments in CQI. The fourth author (NA) is a certified lawyer, medical doctor and PhD student researching accreditation systems. At the time of this evaluation, there were no conflicts concerning CQI that might have influence their opinions.

We chose to use a set of quality criteria for government oversight that did not originate from the field of medical education, because a firm theoretical background in the accreditation literature for medical education was not available.

Our author team was assigned to the roles of both descriptor of the accreditation cases and evaluator of CQI based on the criteria. The authors discussed and evaluated each system using the criteria and searched for patterns with respect to the quality criteria. During these discussions, detailed questions about each system were answered by the author responsible for that system. This process was repeated iteratively until the authors reached consensus for each criterion.

### Case examples: three accreditation systems

In the next section, the accreditation systems are briefly introduced to give the reader a context for our discussion about the pros and cons of CQI. A comprehensive description of the systems may be found on the websites of the accreditation authorities (afmc.ca; cacms-cafmc.ca; lcme.org; www.knmg.nl; www.royalcollege.ca) or obtained from the authors. LP, CC and FS provided detailed descriptions of the systems—both written and oral.

#### The American and Canadian undergraduate accreditation systems

In North America, two authorities accredit medical degree-granting programs: the Committee on Accreditation of Canadian Medical Schools (CACMS) for medical schools in Canada, and the Liaison Committee on Medical Education (LCME) for medical schools in the United States and Canada. The CACMS and the LCME are external bodies with respect to the programs they accredit. The accreditation cycle includes an on-site visit by a peer-review team; the interval between one full survey and the next is 8 years. The internal review process for the full survey requires approximately 2 years and involves a major expenditure of human and financial resources. In 2009, the Association of Faculties of Medicine of Canada (AFMC) approved a mandatory interim review process (IRP) for Canadian medical schools that includes a review of CACMS accreditation standards at the mid-point of the formal accreditation cycle, along with CQI to achieve and maintain compliance with standards throughout the cycle [8]. The AFMC IRP is currently independent of the CACMS, and the effectiveness of the process is the subject of ongoing review. The LCME, with the endorsement of CACMS, has introduced an accreditation standard requiring schools to have a CQI process to monitor compliance with accreditation standards; this came into effect during the 2015–16 academic year [5]. Research is needed to determine the efficacy of these mandatory requirements for CQI.

#### The Dutch postgraduate accreditation system

In the Dutch system there are two important external authorities for the accreditation of residency training: the CGS (Legislative College for Accreditation of Residency Training) and the RGS (Specialist Registration Committee). These are bodies of the Royal Dutch Medical Association (KNMG). The CGS is responsible for setting regulations (training requirements) for accreditation, while the RGS’s role is to monitor compliance with these regulations. The medical specialties in teaching hospitals are visited every 5 years by a survey team commissioned by the RGS. There are no specific mandatory internal quality management systems regarding residency training in teaching hospitals; however, sufficient instruments and systems are made available to conduct internal quality control. The postgraduate medical education programs are required to perform some form of CQI.

#### Continuous professional development accreditation in Canada

There are two basic types of continuous professional development (CPD) accreditation systems: provider-based accreditation and activity-based accreditation. In activity-based CPD accreditation systems, providers submit evidence to the accreditation organization about whether or not an activity they have developed complies with established administrative, educational, and ethical standards. If the accreditation authority agrees that the standards have been met, the activity is approved. Activity-based accreditation is used, for example, by the College of Family Physicians of Canada. Provider-based CPD accreditation systems recognize organizations as institutional providers of continuing education on the basis of their ability to demonstrate adherence to a set of standards. The accreditation process frequently includes an on-site visit in which a peer-review team has the opportunity to discuss specific standards with, among others, the CPD organization’s leadership, administrative staff, and participants. Once an organization is approved, all activities it develops during its accreditation cycle are automatically approved for credit. A provider-based accreditation system is used by the Royal College of Physicians and Surgeons of Canada (RCPSC) to focus on national and provincial CPD organizations, including national specialty societies, while the Committee on Accreditation of Continuing Medical Education (CACME) focuses on the university offices of continuing medical education (CME) in the faculties of medicine. These two provider accreditation systems will be used to illustrate the process by which continuous accreditation is expressed and promoted.

### Criteria for oversight

Accreditation is actually a method of oversight. Oversight functions of government and accreditation processes in medicine may struggle with similar difficulties in achieving quality improvement [20, 21], even though different stakeholders are involved. The Netherlands Scientific Council for Government Policy (WRR) identified seven criteria as the basis for societally responsive oversight [22] (Table 1). Given their general applicability, we used these criteria to evaluate the pros and cons of CQI in undergraduate medical education in the United States and Canada; postgraduate medical education in the Netherlands; and CPD in Canada.

**Table 1:** Summary of the seven criteria and associated tasks

Criteria	Associated tasks
**1. Serve the public interest**	• Take a goal-directed approach with social responsibility as the guiding principle. • Define the ultimate purpose of accreditation and arrange, change, and implement the accreditation standard or system accordingly, without losing the focus on societal needs. Let accreditation standards express the importance of public interests. - Accreditation serves a social function, safeguarding public interests and improving public trust. If accreditation does not emphasize the public interest, the focus on societal needs will be lost. - Analyzing stakeholders and their interests can contribute to this process. Accreditation is typically conducted in a complex environment with stakeholders who have different interests (e.g., representatives of society, program administrators, clinical teachers, trainees, and patients).
**2. Evaluate the benefits**	• Conduct research on the effects of accreditation methods and systems to ensure their legitimacy. • Weigh financial and social benefits against corresponding costs and burdens. • Support the use of evaluation and scientific evidence.
**3. Examine the governance structure**	• The existing governance structure and its entire field of influence should be examined. This should include: - societal forces: key stakeholders and their relationship with the accreditor, the position of the accreditor, existing checks and balances in the field (e.g., at the teaching site) - behavioural mechanisms and incentive structures (e.g., intrinsic motivation, social norms, perceived punitive risks) - constructive interaction with other forms of regulation (e.g., internal/local, international, public, private)
**4. Enhance reflection**	• Have systems in place to alert stakeholders to developments and emerging risks and problems relevant to the public interest. • Share knowledge and provide feedback proactively.
**5. Maintain impartiality and independence**	• Independence is not an end in itself, but supports the impartiality of the accreditor. • Impartiality is important for legitimacy and societal trust.
**6. Be publicly accountable**	• Ensure public accountability for the resources deployed and the outcomes achieved. • Improve communication channels to empower members of the public, institutions, and teaching sites.
**7. Balance expectations with capacity**	• Because the financial and human resources of accreditors are limited, it is important to foster realistic expectations on the part of the accredited parties. • Consider way to distribute costs (e.g., accredited teaching sites can provide financial support for the accreditation process).

## Results

The results are summarized in Table 2 and reflect the authors’ evaluation of the pros and cons of CQI at the institutional level.

**Table 2:** CQI at the institutional level

Criteria	Continuous quality improvement at institutional level	
	Pros	Cons
1. **Serve the public interest**	Aspects reviewed within CQI such as patient safety, quality of education, training, and health care may contribute to safeguarding the interests of society.	
2. **Evaluate the benefits**		In our cases, CQI is not associated with formal cost–benefit evaluations. CQI may introduce an administrative burden and a major expenditure of human and financial resources. Moreover, checks may reveal issues that, when addressed, appear difficult to change, leading to frustration on the part of staff and management.
3. **Examine the governance structure**	Through a requirement for CQI, accreditation authorities can stimulate organizations and their professionals to assume this responsibility. As such, CQI can be a decentralized aspect of the governance structure of the accreditation authority.	CQI at the institutional level may have overlap with other quality systems. Besides excessive bureaucracy, this may lead to conflicting interests and criteria.
4. **Enhance reflection**	CQI can detect risks or problems earlier and enable the timely sharing of feedback and remediation within an organization. The process may be more focused and rigorous given the foreknowledge of staff in the organization about actual or potential risks and problems that need to be addressed.	An ‘open culture’ in which problems are not taboo subjects and in which professionals are encouraged to speak up is necessary for enhanced reflection and subsequent action.
5. **Maintain impartiality and independence**	–	Impartiality and independence in decision making are difficult to attain within an organization. Review processes are conducted by colleagues, and the risk of dependency is much higher within an organization than with reviewers from an external organization.
6. **Be publicly accountable**	–	The institutions adhering to the systems we evaluated do not use CQI to achieve public accountability. In general, organizations do not often communicate the results of their CQI with external audiences.
7. **Balance expectations with capacity**	If CQI is carried out with integrity and provides optimal information to the accreditation authority, the expectation is that the accreditation authority will need less capacity.	–

## Discussion

Our findings show that an integrated system for accreditation of medical education may consist of external authorities and the local CQI system supporting timely reflection and action to serve the public interest. External authorities might even need less capacity due to local quality systems. The downside of CQI is the bureaucracy and costs involved, the risk of interference with other quality systems, the need for an open culture, and potential issues with impartiality and dependency of colleagues involved.

Our findings partially conflict with other literature. Since the 1980s CQI has been used in manufacturing and engineering, the financial sector, education, and health care. Various methods have been used to pursue CQI (e.g., the plan-do-check-act or plan-do-study-act cycle, the Six Sigma standard in manufacturing and other fields, “total quality management”) [23]. All of these share the concept of a “cycle of improvement.” In the manufacturing industry, CQI has been useful in reducing defects, lowering costs, and increasing quality and productivity [24, 25]. In the construction industry it has led to economic benefits, increased customer satisfaction, a reduced need to re-do work, and declining defects in housing projects [26, 27]. Further, CQI is readily applicable to standardized and repetitive processes [23]. In surgical care it has resulted in reduced infection rates and shorter delays in the operating room [23]. Comparing these positive literature reports with the evaluation of CQI in our contexts suggests a publication bias of positive results. It is difficult to measure economic effects and other outcomes in health care and medical education [28–30]. The cost–benefit ratio in our contexts may not always be positive. The difficulty in going through all the components of the plan-do-study-act cycle has been described before [30–32]. The bureaucracy and costs involved in CQI, the risk of interference with other quality systems, the need for an open culture, and potential issues with impartiality and dependency of colleagues involved are important findings. The criteria for oversight used in our analysis were instrumental to the detection of these issues.

Future research addressing CQI in medical education may address design variations [33], elaborate on various perspectives on the cost–benefit evaluation in different contexts [34], and quantitatively measure [35] the effects of CQI in medical education contexts. The gathering of evidence about accreditation systems and CQI in medical education seems to be still in its infancy [5, 9, 36].

The strength of this work is the use of criteria for oversight that provided a lens suitable to critically evaluate the use of CQI. The evaluation gains validity by using cases throughout the continuum of medical education and from three different countries. The cases warrant a naturalistic approach connected to real-life experiences. The evaluation would gain more reliability through the introduction of additional evaluators and cases. Moreover, a shift from qualitative research to a mixed methods design with an attempt to introduce quantitative elements would enhance the value of this kind of research.

## Conclusion

We performed a utility-based evaluation in the hope of reducing complexity in decision making about the use of CQI. The result is one of both pros and cons. The introduction of CQI in the three cases studied may be regarded as an experiment. Further research is needed to find out the contexts in which itis worth investing in CQI. The use of criteria for oversight helped identify issues that will facilitate decision making around the introduction of CQI.

## Data Availability

The data in this study were collected mainly through the expertise and involvement of the authors FS, LP, and CC in accreditation activities and partly from the websites of the accreditation authorities. Because of our reliance on the authors’ expertise, we have provided an extensive description of their professional activities below.

## Abbreviations

AFMC: Association of Faculties of Medicine of Canada
CACME: Committee on Accreditation of Continuing Medical Education
CACMS: Committee on Accreditation of Canadian Medical Schools
CFPC: College of Family Physicians of Canada
CGS: Legislative College for Accreditation of Residency Training
CPD: Continuous Professional Development
CQI: Continuous Quality Improvement
IRP: Interim Review Process
KNMG: Royal Dutch Medical Association
LCME: Liaison Committee on Medical Education
RCPSC: Royal College of Physicians and Surgeons of Canada
RGS: Specialist Registration Committee
